# Challenges in the Diagnostic Performance of Parasitological and Molecular Tests in the Surveillance of African Trypanosomiasis in Eastern Zambia

**DOI:** 10.3390/tropicalmed6020068

**Published:** 2021-04-30

**Authors:** Gloria M. Mulenga, Boniface Namangala, Kalinga Chilongo, Chrisborn Mubamba, Kyoko Hayashida, Lars Henning, Bruce Gummow

**Affiliations:** 1 College of Public Health Medical and Veterinary Services, James Cook University, Townsville, QLD 4814, Australia; mmukuka2000@yahoo.com (G.M.M.); larshnig@jcu.edu.au (L.H.); 2 Department of Veterinary Services, Ministry of Fisheries and Livestock, Lusaka 10101, Zambia; kchilongo@yahoo.co.uk (K.C.); chrisbornmw@yahoo.com (C.M.); 3 Department of Veterinary Services, Kakumbi Tsetse and Trypanosomiasis Research Station, P.O. Box 70, Mfuwe 10101, Zambia; 4 Institute of Distance Learning, The University of Zambia, Lusaka 10101, Zambia; b.namangala@unza.zm; 5 Research Centre for Zoonosis Control, Hokkaido University, Sapporo 0010020, Japan; kyouko.hayashida@gmail.com; 6 Faculty of Veterinary Science, University of Pretoria, Pretoria 0028, South Africa

**Keywords:** diagnosis, African trypanosomiasis, rural areas, Zambia

## Abstract

African animal trypanosomiasis (AAT) control programs rely on active case detection through the screening of animals reared in disease endemic areas. This study compared the application of the polymerase chain reaction (PCR) and microscopy in the detection of trypanosomes in cattle blood in Mambwe, a rural district in eastern Zambia. Blood samples were collected from 227 cattle and tested for infection with trypanosomes using microscopy and Ribosomal RNA Internal Transcribed Spacers (ITS)-PCR. Microscopy on the buffy coat detected 17 cases, whilst thin and thick smears detected 26 cases and 28 cases, respectively. In total, microscopy detected 40 cases. ITS-PCR-filter paper (FP) on blood spots stored on FP detected 47 cases, and ITS-PCR-FTA on blood spots stored on Whatman FTA Classic cards detected 83 cases. Using microscopy as the gold standard, ITS-PCR-FTA had a better specificity (SP) and sensitivity (SE) (SP = 72.2%; SE = 77.5%; kappa = 0.35) than ITS-PCR-FP (SP = 88%; SE = 60%; kappa = 0.45). The prevalence of *Trypanosoma brucei s.l*. was higher on ITS-PCR-FTA (19/227) than on ITS-PCR-FP (0/227). Our results illustrate the complexities around trypanosomiasis surveillance in rural Africa and provide evidence of the impact that field conditions and staff training can have on diagnostic results, which in turn impact the success of tsetse and trypanosomiasis control programs in the region.

## 1. Introduction

Tsetse-transmitted trypanosomiasis, caused by protozoan parasites of the genus *Trypanosoma,* affects both humans and animals. While *Trypanosoma congolense, Trypanosoma vivax* and *Trypanosoma brucei s.l.* cause nagana or African animal trypanosomiasis (AAT) in livestock, the two subspecies of *T. brucei s.l*.: *Trypanosoma brucei gambiense* and *Trypanosoma brucei rhodesiense* are responsible for Human African trypanosomiasis (HAT), commonly known as sleeping sickness. Countries affected by nagana have continued to suffer from economic losses in millions of dollars [1,2,3,4]. The Food Agriculture Organisation (FAO) estimates that 50 million heads of cattle are at risk of AAT with 3 million cattle deaths recorded per year. Loss in cattle production alone is estimated at US$1.0–1.2 billion per year and US$4.7 billion per year in agricultural gross domestic products [4].

Microscopy has been traditionally regarded as the gold standard in detecting the presence of trypanosomes. Microscopic examinations of the buffy coat and wet blood films, as well as thin and thick blood smears stained with Giemsa, are the most common methods used in Africa for trypanosome detection. Microscopy is considered a good diagnostic method because it is simple, cheap and can also simultaneously detect other haemoparasites (microfilaria and *Plasmodium* spp.) [5]. However, microscopy has a very low sensitivity, especially in detecting early infections that are associated with low parasitaemia [6,7,8]. 

Molecular techniques such as the polymerase chain reaction (PCR) have significantly improved the level of sensitivity and accuracy in trypanosome diagnosis in comparison to traditional parasitological methods. However, most remote areas of Africa do not have the resources to facilitate the use of such molecular techniques [9,10]. Molecular tests have the ability to differentiate trypanosome species and subspecies through the use of specific primers [11,12,13,14]. Ribosomal RNA Internal Transcribed Spacers (ITS)-PCR can be used for the detection of both AAT and HAT, but its use in rural settings of Africa is limited by high costs and the need for trained personnel [15,16]. 

Understanding the capabilities of each diagnostic technique is key to the quick and accurate detection of trypanosomes in samples and is critical to disease surveillance, control and eradication. Unfortunately, in most rural settings in Africa, poor detection of trypanosome infections has occurred due to a poor understanding of the limitations of the diagnostic tests used, which can lead to incorrect decision making. Against this background, this study compared the diagnostic performance of microscopy and ITS-PCR in detecting trypanosomes under common field conditions in rural Zambia. 

## 2. Materials and Methods

### 2.1. Study Area

The study was undertaken in Mambwe, a rural district in the eastern province of Zambia from February to April 2019. The district was purposively selected because it is highly tsetse infested and has a high prevalence of bovine trypanosomiasis [17]. Located along the Luangwa river basin, the district covers an area of 4480 km^2^ and is home to the South Luangwa National Park. With a human population of 92,445 belonging to 18,489 households, most of the local community relies on tourism and small-scale farming for their livelihoods (Zambia Central Statistical Office (CSO), 2015).

### 2.2. Study Design and Sample Size

The study compared two diagnostic techniques (ITS-PCR and microscopy) for the detection of bovine trypanosomiasis under rural, field conditions. To facilitate the comparison under field conditions, a trypanosomiasis prevalence survey was conducted using 227 cattle from 193 cattle-owning small-scale farmers in selected parts of the Mambwe district, i.e., located in tsetse-infested parts of the district close to the South Luangwa National Park. The cattle farmers were purposively selected, but their inclusion in the study was largely based on their willingness to participate. Written informed consent from each farmer was required prior to their participation in the survey.

### 2.3. Sample Collection

From each animal, blood was drawn into three capillary tubes (Kimble Chase Life Science, Vineland, NJ, USA) containing heparin (anticoagulant) after puncturing the ear vein of the animal with a blood lancet. One capillary tube was sealed with a crista seal for an on-site examination by buffy coat technique. About 50 µL of blood from the second capillary tube was used to make thin and thick smears for a later microscopic examination at the laboratory [5]. About 50 µL of blood from the third capillary tube was applied onto a well-labeled Whatman FTA Classic Card (GE Healthcare, Madison, WI, USA) and on Whatman^®^ No. 1 filter paper (GE Healthcare). After air drying, both the filter paper and FTA card samples were separately packed in zip locked storage bags containing silica gel and transported to the laboratory for further processing with ITS-PCR [18].

### 2.4. Application of Diagnostic Tests

#### 2.4.1. Microscopy

To increase the chance of parasite detection, three slides were prepared from one animal, i.e., buffy coat, thin and thick smears. An on-site microscopic examination was conducted on cattle blood stored in heparinized capillary tubes. The sealed capillary tubes were spun on-site in a microhematocrit centrifuge for five minutes at 10,000 rpm [19], after which packed cell volumes (PCVs) were determined using a PCV reader. The buffy coat from each sample was then placed on a microscopic slip with a cover slip and examined on site at a ×400 magnification for the presence of motile trypanosomes. At the laboratory, thin and thick smears were stained with Giemsa solution and later examined by trained veterinary laboratory technicians for the presence of trypanosomes [20,21].

#### 2.4.2. DNA Extraction from Whatman^®^ No. 1 Filter Paper

DNA from stored blood spots was extracted using the buffer technique [22]. Two discs of about 3 mm diameter were punched from each blood spot and placed in labeled 1.5 mL sterile tubes. About 66 µL of TE buffer (10 mM Tris-HCl pH 8.0 and 0.1 mM EDTA in distilled water) was added to each tube and incubated at 50 °C for 15 min. The discs were then pressed gently to the bottom of the tube using a new rod for each tube and heated at 97 °C for another 15 min to eluate the DNA. The tubes were then spun down at 10,000 rpm for 1 min [22].

#### 2.4.3. DNA Extraction from FTA Cards

DNA was extracted from the stored blood spots using the Chelex method [18]. Two discs of about 3 mm diameter from each blood spot were placed in a labeled 1.5 mL sterile tube. About 200 µL of Whatman purification reagent was used to wash each disc for 15 min, after which the solution was carefully decanted. The discs were then washed twice with 200 µL of 1% TE buffer for 15 min, after which the solutions were gently decanted. A separate rod for each sample was used during decanting to make sure that the discs did not flow over with the solutions. The discs were then left to air dry for one hour, after which 100 µL of 5% (*w*/*v*) Chelex-100 (Sigma-Aldrich Japan, Tokyo, Japan) in distilled water solution was added and mixed thoroughly. The discs containing Chelex solution were finally incubated at 90 °C for 30 min to elute DNA. The eluted DNA was stored at 4 °C for use within 12 h and at −20 °C for use after 12 h [18].

#### 2.4.4. ITS-PCR

ITS-PCR was undertaken in 25 µL reaction mixtures containing primers AITS-F: CGGAAGTTCACCGATATTGC and AITS-R: AGGAAGCCAAGTCATCCATC [23], One Taq 2 master mix (New England BioLabs, Ipswich, MA, USA), nuclease free water and 5 µL of extracted DNA sample. For the detection of *T. b. rhodesiense*, SRA F (5′-ATAGTGACAAGATGCGTACTCAACGC-3′) and SRA R (5′-AATGTGTTCGAGTACTTCGGCACGCT-3′) were used (procured from Inqaba Biotec, Pretoria, South Africa). Thermocycler amplification conditions were at 94 °C for 5 min, followed by 40 cycles of 94 °C for 40 s, 58 °C for 40 s, 72 °C for 90 min and 72 °C for 5 min. ITS-PCR targets the internal transcribed spacer 1 of the ribosomal RNA (100–200 copies per genome), producing different sized products for different trypanosome species [12,16,23]. ITS-PCR products were separated by electrophoresis (95 volts for 60 min) in a 2% (*w*/*v*) agarose gel containing ethidium bromide. The separated products were then visualized under ultraviolet light in a transilluminator. Known positive controls of *T. congolense* (560–705 bp), *T. vivax* (226–238 bp) and *T. brucei* (415–431 bp) and a negative control were included in each reaction. All samples that were positive for *T. brucei* were subjected to a multiple PCR using a serum resistance-associated antigen (SRA) targeting primer for the detection of *T. b. rhodesiense.*

### 2.5. Data Analysis

Statistical analyses were performed in SPSS version 26 (IBM Corporation, Armonk, NY, USA, 2019). Trypanosomiasis prevalence determined by microscopy (buffy coat, thin smears and thick smears) was used as the gold standard. The prevalence values determined by ITS-PCR were compared against this gold standard, and the sensitivity and specificity were calculated on this basis. A Chi-square test was used to determine the statistical significance between the tests. For expected values under 5, Fisher’s exact test was used. *p* values under 0.05 were considered statistically significant. The impact of the diagnostic test performance was estimated using positive and negative predictive values for each test. The usefulness and benefits between the tests were measured using the ROC, while the kappa coefficient was used to measure agreements and accuracy between tests. The area under the receiver operator curve (AUC-ROC) scores were used to distinguish between a perfect and worthless test. AUC scores were classified as follows: excellent (0.90–1), good (0.80–0.90), fair (0.70–0.80), poor (0.60–0.70) and worthless (0.50–0.60). The kappa values were classified as follows: values ≤ 0 indicated no agreement, slight agreement (0.01–0.20), fair (0.21–0.40), moderate (0.41–0.60), considerable (0.61–0.80) and perfect (0.81–1.00).

## 3. Results

The microscopic examination of trypanosome infection on the buffy coat detected 17/227 cases (7.5%; 95% CI = 4.1–10.9), that on thin smears detected 26/227 cases (11.5%; 95% CI = 7.3–15.6), while that on thick smears detected 28/227 cases (12.3%; 95% CI = 8.1–16.6). Combined microscopy using these three microscopic techniques in parallel recorded a total of 40/227 cases (17.6%; 95% CI = 12.7–22.6). 

Out of the 227 cattle blood samples screened using ITS-PCR (Table 1), the overall prevalence of trypanosomes from blood spots stored and transported on FP was 20.7% (47/227; 95% CI = 15.4–26.0), while a 36.6% (83/227; 95% CI = 30.3–42.8) prevalence was recorded from blood spots stored and transported on FTA cards. The Mean Packed Cell Volume (PCV) for trypanosome positive samples was 34.21 (95% Cl = 33.21–35.22), while that for negative samples was 35.21 (95% CI = 34.21–36.22).

The diagnostic accuracy, sensitivity and specificity of ITS-PCR on blood spots stored on filter paper FP (Accuracy = 0.8; SE = 60%; SP = 87.7%, kappa = 0.45) and those of ITS-PCR on blood spots stored on FTA cards (Accuracy = 0.7; SE = 77.5%; SP = 72.2%; kappa = 0.35) were determined using microscopy as the gold standard. Agreement between the tests was measured using the kappa test. The results of the comparison of ITS-PCR using FTA and FP as the collection method showed an accuracy of 0.69, kappa = 0.27 and *p* value < 0.05, indicating that the difference in the two collection methods was statistically significant.

**Figure 1:**
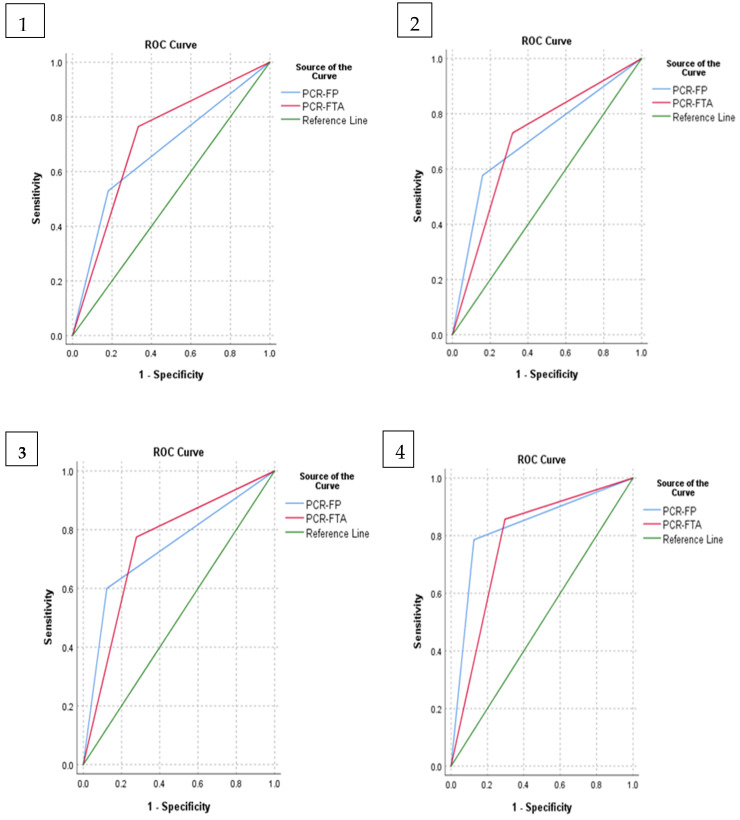
Receiver Operator Curves illustrating the diagnostic abilities of the laboratory tests used. **1**. ITS-PCR compared to buffy coat; **2**. ITS-PCR compared to thin smear; **3**. ITS-PCR compared to think smear; **4**. ITS-PCR compared to combined microscopy.

Receiver operating characteristic (ROC) curves (Figure 1) were used to compare the sensitivity and specificity across a range of values, and the area under the ROC was used to measure the test performance. The curves show the usefulness of ITS-PCR and its ability to detect trypanosomes when compared with the buffy coat (ROC 1), thin smear (ROC 2), thick smear (ROC 3) and combined microscopy (ROC 4).

The AUC-ROC scores for ITS-PCR-FP and ITS-PCR-FTA are shown in Table 2. The higher the AUC score, the better the test at distinguishing diseased from nondiseased individuals.

## 4. Discussion

Our study confirmed that prevalence can be underestimated by a single microscopy technique as compared to combined microscopy methods, while molecular techniques significantly improve the apparent prevalence. Differences and discrepancies in the number of cases detected from the three microscopy tests may be attributed to the climatic conditions under which these tests were conducted, the low parasitaemia of trypanosome species and the time during which observations were made. The use of the buffy coat is considered to be more sensitive than that of thick and thin smears (26), but in this case the buffy coat detected the least number of trypanosomes. An on-site low case detection on the buffy coat can occur when the field conditions do not allow for a thorough screening of samples as compared to a laboratory screening where operators take time to thoroughly screen the samples. Factors that can negatively affect case detection on the buffy coat include poor quality capillary tubes and high ambient temperatures in the study area, which could lead to a diminished motility and/or death of trypanosomes before examiners could observe trypanosome movement in the buffy coat. Other factors include examiners’ inability to observe immature trypanosome movements. 

To validate available molecular diagnostic techniques for AAT, ITS-PCR was employed using blood spots that were stored and transported on FTA cards and normal filter paper. ITS-PCR-FP had a low detection rate compared to ITS-PCR-FTA, which detected a higher number of trypanosomes. This result suggested that blood spots collected and stored on FTA paper were more reliable in determining trypanosome prevalence than blood spots collected and stored on common filter paper (Chi-square *p*-value < 0.01). Such results may be attributed to the fact that FTA paper, unlike common filter paper, has the ability to protect DNA from degradation [18,24].

Unfortunately, due to costs attached to the use of FTA cards, their use may be limited as they may not be readily available to most researchers in trypanosomiasis endemic areas of Africa. The comparative analysis between the use of FTA and FP for blood sample storage and ITS-PCR analysis did, however, show a fair agreement between the two techniques (kappa = 0.27). Our data confirm that both techniques could be useful in the transport of samples for the detection of African trypanosomiasis considering that the transportation of whole blood samples for ITS-PCR analysis may not be feasible under remote field conditions. Our study has demonstrated the convenience of using dry blood samples in areas with limited refrigeration facilities. Practically, dry blood samples could be collected from selected animals and stored on FTA cards or FP on a regular basis for onward analysis at diagnostic centers [7,25]. Both FTA cards and FP may, however, inhibit ITS-PCR, making it less accurate compared to when DNA is extracted directly from whole blood samples, which could explain why microscopically positive samples tested negative on the ITS-PCR test [24].

When the ITS-PCR-FTA results were compared to microscopy, the results indicated a gradual increase in both sensitivity and specificity, with the single microscopy tests reporting the lowest sensitivity and specificity when compared to the combined microscopy tests, which, as expected, had a relatively higher sensitivity and specificity. This pattern of a gradual increase in the ability of the tests to correctly determine infected and noninfected cases was also observed for the microscopy and ITS-PCR-FP comparisons. Such results indicate the need for combining the buffy coat, thin and thick smear techniques when considering microscopy for trypanosome case detection in remote areas of Africa due to limitations in using molecular tests. 

When using the “rule-in” and “rule-out” test, as described by Florkwoski [26], the results showed that ITS-PCR-FTA (kappa = 0.18) (high NPV and high sensitivity) was a better test for identifying diseased cattle than ITS-PCR-FP (kappa = 0.30). AUC-ROC scores for both ITS-PCR-FP (0.7) and ITS-PCR-FTA (0.8) were, however, within the acceptable range of 0.7 to 0.9, indicating that both techniques were acceptable in trypanosome case diagnosis [17,27].

The sourcing, cost and transportation of molecular requirements to perform ITS-PCR was another challenge experienced in this study. Although reagents were available from regional suppliers, the cost was high due to the exchange rate and depreciation of the local currency. Since Zambia does not produce any molecular reagents, importation and transportation costs are a constraint. The use of ITS-PCR is therefore still limited, as most rural laboratories in Zambia have not yet transitioned to the use of molecular techniques for the point of care diagnosis of African trypanosomiasis and other zoonotic diseases. Findings from this study highlighted the limitations of the existing tests for African trypanosomiasis in rural areas of Africa, i.e., microscopy and ITS-PCR, which may have crucial clinical and epidemiological implications [7,16,28]. The mAECT (mini Anion Exchange Centrifugation Technique) was not used in this study area and could be a technique worth considering in the future in order to improve sensitivity [29].

Although previous studies suggested that *T. congolense* was the main cause of AAT and anaemia in Eastern and Southern Africa [7,30,31,32], data from the current study demonstrated that *T. vivax* was present in most of the sampled cattle and that anaemia was not an indicator for trypanosome infection. ITS-PCR has previously been reported as being better at detecting *T. vivax* infections when compared to other trypanosome species [9,16], which may partially explain their high prevalence in these results. The high prevalence of *T*. *vivax* infections may also suggest that trypanosomiasis transmission within the sites included in this study could be mechanical by other blood sucking insects, such as tabanids, prevalent in the area [33] rather than by tsetse flies. 

Finally, the detection of the human infective trypanosomes *T. b. rhodesiense* from cattle blood samples analyzed in this study highlights the risks that cattle pose to communities living in tsetse-infested areas [1]. Cattle may be potential sources of sleeping sickness when humans get bitten by tsetse after the fly has taken a blood meal from an infected animal [34,35,36]. Our results support the need for a more holistic approach in the control of trypanosomiasis with a focus on the control of the disease in domestic animal reservoirs.

## 5. Conclusions

This study serves as a prime example of the impact that remote field conditions and staff training can have on results that in turn impact the success of tsetse and trypanosomiasis control programs in the region. The study illustrates current challenges with AAT diagnosis using molecular and microscopy techniques in rural areas and the need for innovation in field diagnostics. However, considering that trypanosomiasis is prevalent in remote rural areas where access to diagnostic facilities is limited, FTA cards and FP should be considered for collecting, storing and transporting blood samples for analysis using ITS-PCR where the collection of whole blood is not feasible. Currently used diagnostic tests have their own advantages and limitations. ITS-PCR is a good screening test of trypanosomes causing nagana. However, the use of ITS-PCR may be limited and impractical in remote rural areas of Africa where trypanosomiasis is endemic. Microscopy could, therefore, be used for diagnosis but as a combination of the three commonly used techniques of buffy coat, thin smears and thick smears. Microscopy remains the most practical option for the diagnosis of trypanosomes in the field, but understanding its limitations is critical when using it for surveillance purposes. Better staff training in disease diagnosis, better maintenance of diagnostic equipment, a better funding model and an improvement in field quality control would help address challenges in disease diagnosis, as highlighted in this study. 

## Figures and Tables

**Table 1:** Prevalence of trypanosome species in cattle (n = 227) by PCR.

Trypanosome Species	PCR-FP	Sample Prevalence %	Confidence Interval at 95%	PCR-FTA	Sample Prevalence %	Confidence Interval at 95%
*T. congolense*	7	3.1	0.8–5.3	14	6.2	3.0–9.3
*T. vivax*	39	17.2	12.3–22.1	50	22.0	16.6–27.4
*T. brucei*	1	0.4	−0.4–1.3	19	8.4	4.8–12.0
*T. b. rhodesiense*	0	−	−	3	1.3	−0.2–2.8
*Mixed*	1	0.4	−0.4–1.3	9	4.0	1.4–6.5
TOTAL	47	20.7	15.4–26.0	83	36.6	30.3–42.8

**Table 2:** Areas under the ROC curves shown in Figure 1.

Reference	Test Result Variable(s)	AUC	Std. Error	*p*-Value	AUC 95% Confidence Interval		Test Performance Relative to Reference
					Lower Bound	Upper Bound	
(1) Buffy coat	ITS-PCR-FP	0.674	0.075	0.020	0.528	0.821	Poor
ROC 1	ITS-PCR-FTA	0.716	0.063	0.001	0.592	0.839	Fair
(2) Thin smear	ITS-PCR-FP	0.709	0.060	0.001	0.591	0.827	Fair
ROC 2	ITS-PCR-FTA	0.706	0.054	0.000	0.601	0.812	Fair
(3) Thick smear	ITS-PCR-FP	0.830	0.047	0.000	0.738	0.922	Good
ROC 3	ITS-PCR-FTA	0.780	0.044	0.000	0.695	0.866	Good
(4) Combined Microscopy	ITS-PCR-FP	0.739	0.049	0.000	0.643	0.834	Fair
ROC 4	ITS-PCR-FTA	0.748	0.043	0.000	0.665	0.832	Fair

## Abbreviations

PCR	Polymerase chain reaction
AAT	Animal African trypanosomiasis
HAT	Human African trypanosomiasis
ITS	Internal transcribed spacers
FP	Filter paper
PCV	Packed cell volume
NPV	Negative predictive value
PPV	Positive predictive value
AUC	Area under curve
ROC	Receiver operator curve
DNA	Deoxyribonucleic acid
